# A resource on latitudinal and altitudinal clines of ecologically relevant phenotypes of the Indian *Drosophila*

**DOI:** 10.1038/sdata.2017.66

**Published:** 2017-05-16

**Authors:** Subhash Rajpurohit, Xiaqing Zhao, Paul S. Schmidt

**Affiliations:** 1 Department of Biology, University of Pennsylvania, 433 S University Ave, Philadelphia, Pennsylvania 19104, USA; 2 Department of Pathology, University of Washington, 1959 NE Pacific Street, Seattle, Washington 98195, USA

**Keywords:** Evolution, Ecology, Entomology

## Abstract

The unique geography of the Indian subcontinent has provided diverse natural environments for a variety of organisms. In this region, many ecological indices such as temperature and humidity vary predictably as a function of both latitude and altitude; these environmental parameters significantly affect fundamental dynamics of natural populations. Indian drosophilids are diverse in their geographic distribution and climate tolerance, possibly as a result of climatic adaptation. These associations with environmental parameters are further reflected in a large number of clines that have been reported for various fitness traits along these geographical ranges. This unique amalgamation of environmental variability and genetic diversity make the subcontinent an ecological laboratory for studying evolution in action. We assembled data collected over the last 20 years on the geographical clines for various phenotypic traits in several species of drosophilids and present a web-resource on Indian-*Drosophila* ( http://www.indian-drosophila.org/). The clinal data on ecologically relevant phenotypes of Indian drosophilids will be useful in addressing questions related to future challenges in biodiversity and ecosystems in this region.

## Background & Summary

Drosophilid flies are classical models for population genetic and evolutionary ecology studies^1–3^. *Drosophilids* as a broader taxonomic group are very diverse in subtropical India, with 25 genera and over 287 species identified to date^4–6^. As in several other taxonomic groups, the genus *Drosophila* is species-rich in India, presumably due to the extensive heterogeneity in ecological conditions. This environmental heterogeneity may have spurred diversification among populations and taxa, and many species in this genus are endemic to the Indian subcontinent^6^.

The Indian subcontinent ranges from 8.4 to 37.6 °N in southern Asia. It is mostly situated on the Indian plate and extends southward into the Indian ocean from the Himalayas. The region is characterized by a diverse geography with an extensive altitudinal range, vast central plains, and large river valleys in the northern foothills of Himalayas. The coefficient of variance of temperature and relative humidity changes predictively from the subtropical lower Himalayas to the tropical southern peninsula. The cumulative effects of the cold northern Himalayas, inland hot plains, and surrounding water bodies are considered as major drivers of the climatic gradients. Further details on these climatic variables can be found in Rajpurohit *et al.*^7^ and Rajpurohit and Nedved^8^.

Phenotypic and genetic clines in various drosophilid species on the Indian subcontinent have been well documented^7,8^. These studies have primarily examined latitudinal and/or altitudinal variation among populations in traits measured under common garden conditions. Some intriguing patterns have emerged, such as opposing clines for desiccation resistance and starvation resistance (reviewed in Rajpurohit and Nedved^8^). The environmental gradients present on the Indian subcontinent, in conjunction with the observed phenotypic and genetic clines, sets a platform for studies of local adaptation to natural variation in environmental conditions. These are also the ideal places to investigate adaptive dynamics of fitness-associated traits^8^. Natural selection acting along environmental gradients could result in the formation and maintenance of such clines^8–10^. Such patterns of association can also reflect aspects of demography^11^; if a clinal pattern is replicated in independent samples (e.g., populations sampled across gradients on multiple continents) the inference of selection is strengthened^12^.

However, studies of wild drosophilid populations in India are not as well-known as similar studies done on European, North American and Australian populations, presumably because of limited circulation or studies published in local journals. Studies from this region are not as well incorporated in the conceptual and empirical understanding of climatic adaptation. Therefore, we assembled data from previous studies on ecologically relevant fitness traits of the Indian drosophilids to generate a comprehensive, one-stop portal for the scientific community. The Indian *Drosophila* cline dataset contains data from ten *Drosophila* species from studies published between 1998 and 2013 and includes traits related to morphological variation, life-histories, stress resistance, behavioral differences and genetic markers. A majority of these studies used common garden experiments to test for latitudinal and/or altitudinal differentiation among populations, thus focusing on variation at the phenotypic level^8^. More recently, studies have also examined range shifts in species boundaries in the context of climate change scenarios^13,14^.

The compilation of historical datasets and species distributions in a central database repository will also be useful for assessing climate change related studies in this region.

To summarize existing studies on phenotypic and genetic clines of the Indian populations of *Drosophila,* and to track shifts in these clines over time, the ‘Indian-*Drosophila*’ portal (http://www.indian-drosophila.org/) was established. The ‘Indian-*Drosophila*’ web-resource contains: (1) latitudinal clinal data (i.e., trait variations along Indian latitudes), (2) altitudinal cline data (trait variations along Indian altitudinal ranges), and (3) details on species taxonomy and distribution. The web-resource will be continually updated and user-friendly interfaces will be incorporated as needed. In the present format of the resource a user-friendly application named DrosoCline has been incorporated for data visualization purposes.

## Methods

### Latitudinal and altitudinal traits in Indian drosophilids: Dataset selection

**Figure 1:**
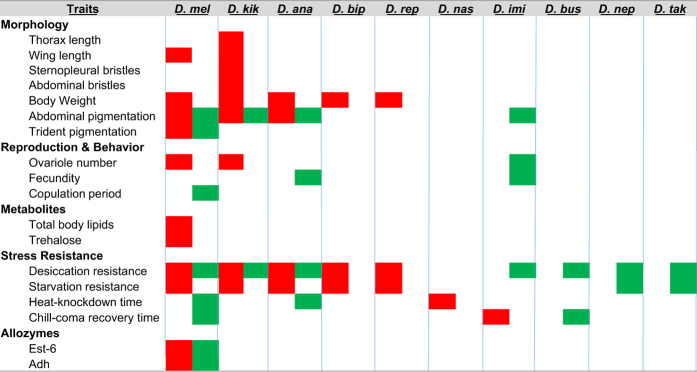
Status of species and traits covered in this study showing latitudinal or altitudinal clines in India. The red and green boxes represent the presence of latitudinal and altitudinal clines, respectively. *D. mel*: *Drosophila melanogaster*; *D. kik*: *Drosophila kikkawai*; *D. ana*: *Drosophila ananassae*; *D. bip*: *Drosophila bipectinata*; *D. rep*: *Drosophila replete*; *D. nas*: *Drosophila nasuta*; *D. imi*: *Drosophila immigrans*; *D. bus*: *Drosophila buskii*; *D. nep*: *Drosophila nepalensis*; *D. tak*: *Drosophila takahashii*.

We began the development of this community resource with an exhaustive search of papers published between 1987 and 2015 that examined clinal variation in Indian populations of various drosophilid species. We selected papers and datasets which conformed to the following criteria: (1) at least three populations were included along the latitudinal/altitudinal transect; (2) traits were measured under common garden conditions, i.e., excluding studies where direct measurements were done on wild collected samples; and (3) if parallel studies of the same cline had been conducted, we selected the first, initial study only. With these criteria 16 papers were selected and incorporated to this web-resource. A point to note, most of the traits in this web-resource exhibited significant clines and were reported accordingly; there is the possibility of some bias, as work that failed to identify significant clines may not have been published. In the present clinal datasets five major categories of traits have been incorporated: (1) morphology, (2) reproduction and behavior, (3) metabolites, (4) stress resistance, and (5) allozymes (see Fig. 1).

**Figure 2:**
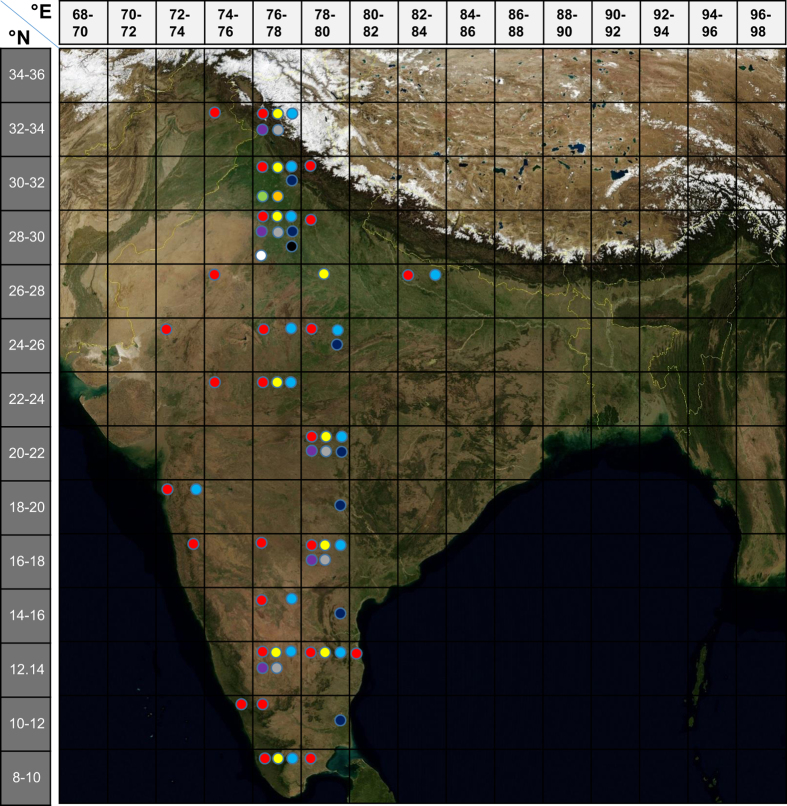
Grid map depicting the distribution of various *Drosophila* species along the Indian latitudes. The exact geographical coordinates and elevation (Latitude, Longitude, and Altitude) of collection sites are provided in Table S3. The ‘Indian-*Drosophila*’ web-resource consists of data on a range of traits showing altitudinal and /or latitudinal variations. Each color dot represents a species. A 2/2 latitude-longitude grid locates the origin of populations used in the various clinal studies. Red: *D. melanogaster*; Yellow: *D. kikkawai*; Light blue: *D. ananassae*; Purple: *D. bipectinata*; Gray: *D. replete*; Dark blue: *D. nasuta*; Light green: *D. immigrans*; Organe: *D. buskii*; Black: *D. nepalensis*; White: *D. takahashii*.

A total of 61 datasets have been incorporated in this resource from which 32 are latitudinal and 29 are altitudinal (see Data Citation 1). These datasets span ten different species, representing the most comprehensive range of studies in drosophilids. This is the only collection of datasets that incorporates both latitudinal and altitudinal clinal data from a single geographical region (the Indian subcontinent). The geographical distribution of populations from which these 61 clinal datasets are derived is between 9–33° N/ 75–83° E (see Fig. 2). This collection of datasets covers a total of 56 collection sites (the origin of sites of different *Drosophila* species populations are given in Fig. 2; also, see Data Citation 1). Latitude, longitude, and altitude of collection sites are provided in Data Citation 1. Climatic data for these sites can be accessed from the India Meteorological Department, Pune (http://www.imdpune.gov.in/).

### Traits values: Standardization

A total of 18 different traits under five major categories have been incorporated in this resource (Data Citation 1). The morphological traits include thorax length, wing length, sternopleural bristle number, body weight, trident pigmentation, and abdominal pigmentation^15–18^. Thorax length was measured from the posterior tip of the scutellum to the anterior margin of the thorax. Wing length measured from the point of attachment with thorax up to the tip of the third longitudinal vein. The thorax and wing measurements are expressed in mm×100. Trident pigmentation was scored by visual examination of dorsal side of the thorax using four phenotypic classes. Abdominal pigmentation was scored by the visual examination of lateral side of the abdominal tergites using discrete phenotypic classes. Body weight is expressed as mg×100.

The category for reproduction and behavior included ovariole number, fecundity and copulation duration^19,20^. Ovariole number was presented as average or sum of both ovary lobes. Fecundity was reported as the number of eggs laid per fly per day. Copulation duration was measured as the time from successful mounting to separation and is expressed in minutes.

The metabolites category included total lipid content as well as trehalose content^21^. For lipid content, the estimation method as described by Marron *et al.*^22^ was followed. Individual flies were dried at 60 °C for 48 h in smaller tubes and weighed. Di-ethyl ether was subsequently added to the samples and agitated at 37 °C for 24 h. Flies were then removed from the solvent and dried again at 60 °C for 24 h and reweighed. Lipid content was calculated by subtracting lipid free mass from dry mass. Trehalose content was estimated using commercially available kits (Megazyme trehalose assay kit). Flies were homogenized in groups and heated in a water bath set at 95 °C for 20 min. Subsequently, samples were centrifuged at 12,000 r.p.m. for 15 min and aliquots of the supernatant were digested with trehalase at 37 °C. The reaction absorbance was measured at 340 nm.

Stress resistance traits included desiccation resistance, starvation resistance, heat-knockdown time, and chill-coma recovery time^23–30^. Desiccation assays were performed in narrow size *Drosophila* culture vials where no food and water was provided. Flies were kept individually or in groups of ten with silica gel desiccant; flies were separated from the silica gel desiccant by a foam plug. To measure starvation resistance, flies were kept in narrow *Drosophila* culture vials in the absence of food. Water was made available with a cotton ball soaked in water or with 1–2% agar media in the vial. For heat-knockdown time measurements, individual flies were placed in glass tubes submerged in a water bath set at 39 ° C. To measure chill-coma recovery time, flies were placed in empty glass vials and buried in ice for a specific period of time. Flies were then brought to room temperature and the recovery time of each individual was recorded.

Adh and Est-6 allozymes were examined for allele frequency variation among populations using standard starch gel electrophoresis and staining methods. Each population was characterized by the frequency of one of the common electrophoretic alleles (for both ADH and Est-6, the frequency of the ‘fast’ mobility allele, F, was reported). For all the traits described above, the original publications give further methodological details. The original data with the accompanying reference can be downloaded using the DrosoCline application.

## Data Records

A total of 61 datasets are made available through the Indian-*Drosophila* web-resource (http://www.indian-drosophila.org/). The datasets can be accessed through the DrosoCline application (https://indian-drosophila.shinyapps.io/DrosoCline/). A direct link for this application is provided in the Indian-*Drosophila* web-resource. DrosoCline allows the user to choose the type of geographical range (latitude or altitude), species, sex, and phenotype. The user can also download the data for the selected combinations by clicking the associated download tab. The downloaded data file (in.csv format) also contains information regarding the published references. This resource currently archives data on latitudinal and altitudinal variations for 18 ecologically relevant traits in 10 species, and more datasets will be incorporated as they become available. We propose to update the data portal via biannual literature surveys and direct author submission.

## Technical Validation

The datasets reported in this collection are already published in peer-reviewed journals^15–21,23–31^. The data presented here in the paper and on the online resource have also been statistically analyzed in the associated published work. Data have been re-visualized and checked for possible typing errors in the various published papers. If any discrepancies were found they were corrected before placing them on the web-resource. Only the experiments that included control treatments and adequate replication were included in this analysis and the associated web-resource.

## Usage Notes

Severe effects of global warming are projected for South Asia. The Regional Climatic Modeling System PRECIS (Providing Regional Climate for Impact Studies) developed by the Hadley Center predicts a 2–4 °C temperature rise in India as the 21st century progresses, with the northern parts of the India being more sensitive to this change^32^. A temperature profile for the past 100 years (1900–2000) on the Indian subcontinent is shown elsewhere (see Fig. 5 in Rajpurohit *et al.*^7^).

The global temperature rise may result in increasing physiological challenges that put organisms, taxa, and entire ecosystems at risk. Recent studies have clearly shown range expansion for many populations, and that such shifts are accompanied by changes in phenology and rapid adaptation to environmental change^26,33,34^. Studies on phenotypic differentiation across environmental gradients could be a valuable tool to understand fundamental aspects of the evolutionary response to climate change. In *Drosophila* species, genetic and phenotypic change across geographical transects could provide insight into the connectivity between climate change and evolutionary shifts. Along these lines, recent work on Indian altitudinal transects in the Western Himalayas has shed light on habitat shrinkage and expansion associated with climate change^13,14^. Ongoing studies from the Indian subcontinent will provide further opportunities for more precise studies merging patterns of phenotypic and genetic variation, species distributions, historical data, and climate change.

This unique amalgamation of taxonomic and genetic diversity, as well as the presence of robust clines for multiple traits across species, makes the Indian subcontinent an ecological laboratory for studying evolution in action. Pimm *et al.*^35^ proposed that information on species distribution and geographical variations over time could provide a basic framework to infer evolutionary responses associated with climate change^35^. The resource we develop here will help the scientific community track spatiotemporal changes in phenotype and associated genomic regions, and can also be used to examine associations between pattern shifts and temporal change in specific climatic variables.

Resources such as the Indian-*Drosophila* Database also allow us to make comparisons across populations residing on different continents. A resource for Australian drosophilids already exists^36^. A cross geographical comparison could be used to explore fundamental and unaddressed questions in *Drosophila* ecology and evolution.

## Additional Information

**How to cite this article:** Rajpurohit, S. *et al.* A resource on latitudinal and altitudinal clines of ecologically relevant phenotypes of the Indian *Drosophila*. *Sci. Data* 4:170066 doi: 10.1038/sdata.2017.66 (2017).

**Publisher’s note:** Springer Nature remains neutral with regard to jurisdictional claims in published maps and institutional affiliations.

## Supplementary Material
